# Prostatic Acid Phosphatase (PAP) Predicts Prostate Cancer Progress in a Population-Based Study: The Renewal of PAP?

**DOI:** 10.1155/2019/7090545

**Published:** 2019-01-23

**Authors:** Huan Xu, Fubo Wang, Huizhen Li, Jin Ji, Zhi Cao, Ji Lyu, Xiaolei Shi, Yasheng Zhu, Chao Zhang, Fei Guo, Ziyu Fang, Bo Yang, Yinghao Sun

**Affiliations:** Department of Urology, Shanghai Changhai Hospital, Second Military Medical University, Shanghai, China

## Abstract

**Objective:**

To characterize the disease progression and median survival of patients with prostate cancer (PCa) according to the prostatic-specific acid phosphatase (PAP) analysis in a population-based study from the Surveillance, Epidemiology, and End Results (SEER) database.

**Materials and Methods:**

Prostate cancer patients with completed PAP results were identified using the SEER database of the National Cancer Institute. The Mann-Whitney Sum test was utilized to compare the statistical significance for measurement data and ranked data. Data were stratified by ages, races, TNM Classification of Malignant Tumors (TNM), pathological grades, number of tumors, PAP, and survival duration. Multivariable logistic analysis was performed to identify predictors of the presence of invasion and metastases. Cox regression was analyzed for the factors associated with all-cause mortality and prostate cancer-specific mortality. Moreover, survival curve was used to detect the survival months. The unknown data were excluded from these tests.

**Results:**

In total, there are 5184 PAP+ patients and 3161 PAP- patients involved. The Mann-Whitney Sum test showed that slightly greater tumor size (*P* = 0.03), elevated lymphatic (*P* = 0.005) and distant (*P* < 0.001) metastasis rate, higher pathological grade (*P* < 0.001), localized tumor number (*P* < 0.001), and shortened survival months (*P* < 0.001) were observed in the PAP+ group compared with the PAP- group. In the multivariable logistic regression, invasion and metastasis Hazard Ratio (HR) were elevated significantly (*P* < 0.001) in the PAP+ individuals. In the survival analysis, PAP- patients experienced the prolonged median survival. In the postsurgical patients, the survival months were still longer in PAP+ patients compared with the negative ones (*P* < 0.001), though surgery prolonged the survival months of both groups. Survival months stratified by localized, invasion, and metastasis situations were analyzed. In the three stratified subgroups, the survival duration is significantly decreased in the PAP+ individuals in the localized PCa group (*P* < 0.001) and the metastasis group (*P* = 0.013).

**Conclusions:**

The findings of this study provide population-based estimates of the PCa progress and prognosis for patients with different PAP results, which may suggest a renewed period for the PAP.

## 1. Introduction

Prostate cancer (PCa) is the second most frequently diagnosed cancer with the fifth mortality in the males [1]. Screening markers, prostate-specific antigen (PSA) included, can result in the early detection of the disease [2]. Nevertheless, the predictive function of this biomarker for the PCa progress is still limited. Robust population-based estimates relating to the progress of PCa, including the metastases, localized tumor numbers, tumor sizes, survival years, and cancer-specific survival (CSS), at PCa are lacking, partly due to the single widely used evaluated index in the clinic.

Human prostatic-specific acid phosphatase (PAP) is a secreted glycoprotein with the molecular weight of 100 kDa synthesized in lysosomes of prostate epithelial cells [3]. It has been determined to be associated with the weight of prostate tissue [4]. There are two forms of PAP, including the cellular form (cPAP, highly expressed in the prostate cells) and the secretory form (sPAP, expressed only in the prostate and is mostly released into seminal fluid) [5], with different isoelectric points and molecular weights [6]. Some reported that different mRNA was encoded in the different forms of PAP and the physiologic substrate is still needed to be studied [6]. The PAP is increased in the circulation of PCa patients while its prostate expression is reduced. As is claimed, cPAP has a growth-suppressing effect and it is due to its cellular protein tyrosine phosphatase activity and both PAP mRNA and protein levels are decreased or absent in prostate carcinoma tissue [7, 8]. As is mentioned, the cPAP is low regulated in the prostate cancer tissue, and this decrease results in the extracellular signal-regulated kinase (ERK)/mitogen-activated protein kinase (MAPK) signaling pathway, leading to the loss of their androgen sensitivity and an increase in the growth rate and tumorigenicity. Furthermore, PAP is reported to block the PI3K-AKT-AR pathway, which elevates the cell survival rate [5, 9]. Moreover, cPAP tends to regulate the growth of the bone metastasis via the alteration of the RANKL/OPG system [10]. In sum, there are two different forms of PAP and the change in the process of prostate cancer is various.

PAP is the most important screening marker in the past decades until the appearance of the PSA detection. After the widely use of PSA as a screening marker, PAP was used less and less. However, the use of the PAP in clinic work is renewed recently, partly because of the use of PAP as the first cancer vaccine. Moreover, more clinical researches have reported the detective function of PAP for the PCa metastases, especially for the bone osteoblastic lesions. It has also been reported that PAP was detected in high Gleason Score prostate cancer [11]. Thus, it is accepted that PAP may be a candidate to be widely used as the detector of PCa progress. However, the population-based data are still lacking.

In this research, we used the Surveillance, Epidemiology, and End Results (SEER) database to characterize the effect of PAP on the PCa progress and the prognosis, especially postsurgical, at the time of cancer diagnosis among patients with PCa on a population-based level.

## 2. Materials and Methods

### 2.1. Population Source

The current study comprised SEER-related data. The SEER database includes information on cancer, which is from the National Cancer Institute (NCI) in the United States (US), for 28% of the US population. In this research, patient information during 1998-2003 was analyzed, during which period the PAP was used as the screening method for PCa and after 2003, PAP is not registered in the SEER database since then.

### 2.2. Study Population

**Figure 1:**
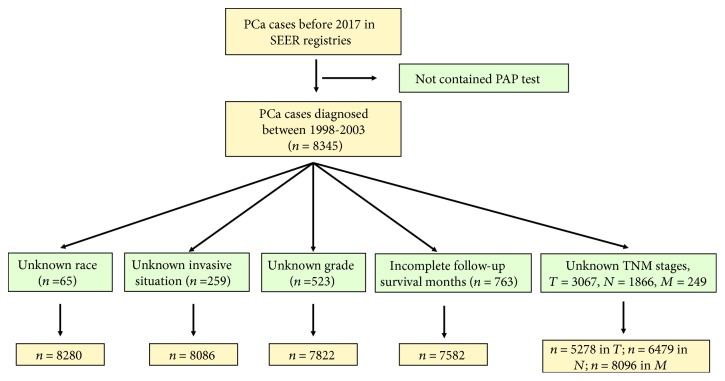


Within the SEER database, we identified 5184 PAP-positive (PAP+) individuals and 3161 PAP-negative (PAP-) individuals diagnosed with prostate cancer (International Classification of Diseases (ICD)). For each subgroup, the following were excluded: individuals with unknown race (*n* = 65), TNM stages (*n* for *T* = 3067, *n* for *N* = 1866, and *n* for *M* = 249), invasive situation (*n* = 259), grade (*n* = 523), and follow-up survival months (*n* = 763), which process is shown in Figure 1.

### 2.3. Covariates

For each subject, age at diagnosis (30-50, 51-70, 71-85, and ≥86), races (white, black, Asian, and American Indian), tumor size (*T*_1−4_), lymphatic metastases (*N*_0_ and *N*_1−3_), distant metastases (*M*_0_ and *M*_1_), invasive status (localized tumor, invasive tumor, and metastasis tumor), pathological grade (grade_1-4_), and number of localized tumors were assigned. Moreover, the following were also analyzed: survival months, prostate cancer-specific survival months, and survival months for the surgical patients. The invasion status was determined by *SEER Extent of Disease Codes and Coding Instructions* 3rd edition. The survival duration was analyzed only for the completed data with the exact endpoint. Surgery status was according to the “Reason no cancer-directed surgery” section. PAP data was gotten from the “tumor marker 1” of the prostate cancer and exact definition was referenced in *SEER Self Instructional Manual for Cancer Registrars* book 5. The nonparametric variable was performed using median (quartiles) and Hazard Ratio was present in the logistics analysis. Hierarchical variable was present by numbers and ratio.

### 2.4. Statistical Analyses

The Mann-Whitney Sum test was utilized to compare the statistical significance of differences in medians and proportions for measurement data and ranked data. To further confirm the results, the multivariable logistic regression was used to determine whether the age and race were associated with the presence of invasion and metastases at diagnosis; other variables in the model included pathological grade and number of localized tumors. The likelihood ratio test (LRT) is used in the multivariable logistic regression analysis. Survival estimates were obtained using the Kaplan-Meier method. The multivariable Cox regression was performed to identify covariates associated with increased all-cause mortality using the same variables as in the logistic regression model described. In the Cox regression model, enter method was used. All tests were two-sided, with a significance level set at ^∗^*P* ≤ 0.05 and ^∗∗^*P* ≤ 0.01.

## 3. Results

The flow diagram for this analysis was shown in Figure 1. The total PAP+ patient number is 5184 and the PAP- number is 3161. After the exclusion, the exact number of each group is shown in Table 1. The demographic and tumor characteristics of the study cohort are also presented in Table 1. The Mann-Whitney Sum test showed that PAP+ patients presented lymphatic metastases more often than the PAP- individuals (*P* = 0.005). As is confirmed in our research, the distant metastases rate is elevated in the PAP+ group (^∗∗^*P* < 0.001), with elevated pathological grade in the PAP+ group (^∗∗^*P* < 0.001). The localized tumor number is more in PAP+ group (^∗∗^*P* < 0.001) though the median level of each group is the same. In the survival analysis shown in Table 1, PAP- individuals present the extended survival months (134.0 (63.0-161.0) vs. 143.0 (88.0-166.0), ^∗∗^*P* < 0.001). For the prostate cancer-specific survival months, it also presented the similar results (47.00 (16.00-90.00) vs. 93.00 (52.75-132.00), ^∗∗^*P* < 0.001). In the analysis of patients who has undergone surgery treatment, the surgery indeed prolonged the survival duration of the PCa patients (*P* < 0.001). However, the PAP+ individuals still presented the shortened survival months (144.0 (95.0-168.0) vs. 152.0 (125.8-172.0), ^∗∗^*P* < 0.001).

The multivariable logistic regression was used for the presence of metastases at diagnosis of PCa shown in Table 2. The pathological grade and the number of localized tumors were significantly related to the metastasis situation (^∗∗^*P* < 0.001). In PAP+ group, invasion (1.78(1.39-2.26)) and metastasis (3.03 (2.49-3.70)) Hazard Ratio (HR) were elevated significantly compared with the negative individuals (^∗∗^*P* < 0.001).

On multivariable Cox regression (Table 3) for all-cause mortality among patients with PCa at diagnosis, T3 stage (vs T1; HR, 1.33 (1.17-1.50); ^∗∗^*P* < 0.001), N1 stage (vs. N0; HR, 2.85 (1.68-4.85); ^∗∗^*P* < 0.001), N3 stage (vs. N0; HR, 3.82 (1.41-10.32); ^∗∗^*P* < 0.001), grade 2 (vs. grade 1; HR, 0.79 (0.65-0.96); ^∗^*P* = 0.02), grade 4 (vs. grade 1; HR 2.39 (1.20-4.73); ^∗^*P* < 0.001), invasive tumors (vs. noninvasive; HR, 2.09 (1.64-2.66); ^∗∗^*P* < 0.001), metastasis tumors (vs. noninvasive; HR, 6.48 (3.42-12.30); ^∗∗^*P* < 0.001), and surgery treatment (vs. nonsurgery; HR, 1.12 (1.00-1.26); ^∗^*P* = 0.05). Prostate cancer-specific mortality among patients with PCa at diagnosis is also presented in Table 3. Interestingly, though no significant changes were observed in the PAP positive and negative in the all-cause mortality group, it is significantly increased in the PCa-specific mortality group (vs. PAP-; HR, 2.87 (2.48-3.32); ^∗∗^*P* < 0.001). For the surgery treatment in PCa-specific mortality group, no significant changes were observed (vs. nonsurgery; HR, 1.09 (0.91-1.30); *P* = 0.36).

**Figure 2:**
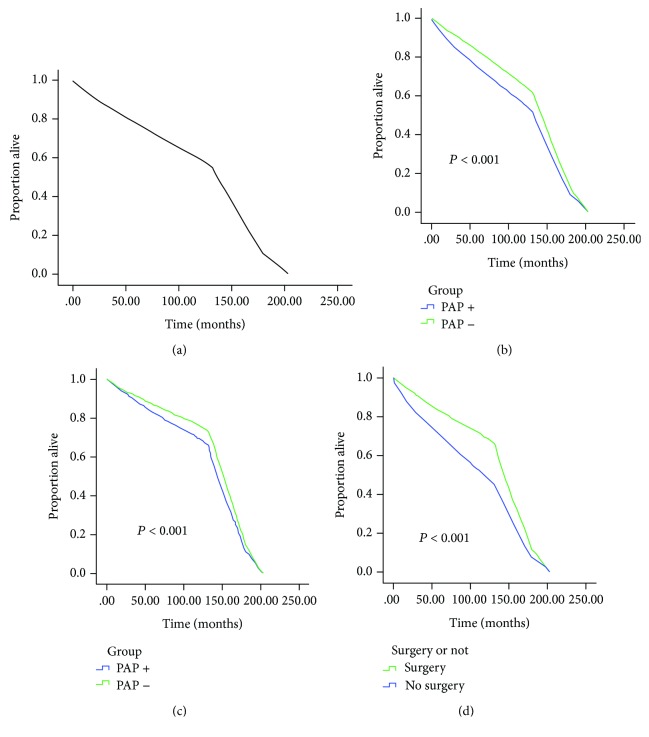


In the survival curve analysis, the median survival among the entire cohort was 136 (69-163) months, with patients with PAP negative subtype experiencing the prolonged median survival (143 (87-167)). In the postsurgical patients, the survival months were still longer in PAP- patients (144 (95-168) vs. 152 (126-172), ^∗∗^*P* < 0.001). The overall survival estimates (Figure 2(a)), as stratified by PAP subtype (Figure 2(b)), and the PAP effect on the survival months after surgical treatment (Figure 2(c)) are graphically displayed in Figure 2. The surgery effect on the survival months in the PAP-positive patients was also studied in Figure 2(d) which showed that surgery prolonged the survival months of the PAP-positive individuals (95 (144-168) vs. 48 (118-156), ^∗∗^*P* < 0.001).

**Figure 3:**
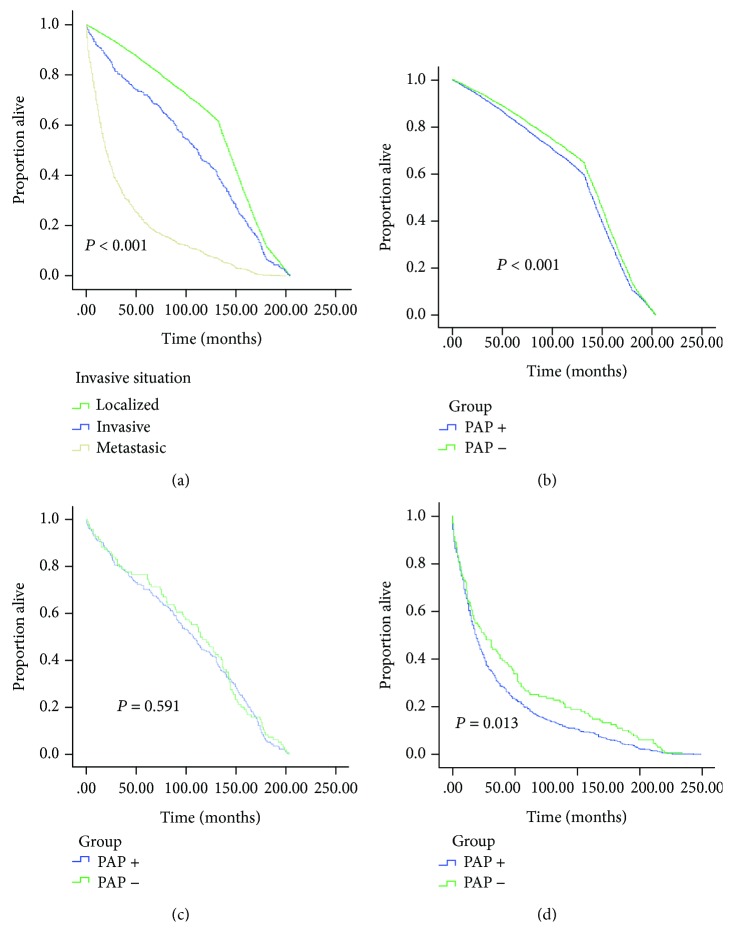


Survival months stratified by localized, invasion and metastasis situations were analyzed in Figure 3(a). In the three stratified subgroups (shown in Figures 3(b)–3(d)), the survival duration is significantly decreased in the PAP+ individuals in the localized PCa group (86 (140-164) vs. 99 (146-168), ^∗∗^*P* < 0.001) and the metastasis group (7 (19-46) vs. 8 (26-69), ^∗^*P* = 0.013).

## 4. Discussion

Prostate cancer screening has been widespread using PSA since 1990s. After that time, PAP was used less and less in the clinical work. This was because PSA was more sensitive than PAP in the serum detection and screening of prostate cancer. The use of PSA, however, also leads to overdiagnosis and overtreatment of prostate cancer. Moreover, it is not effective to use in the prediction of the metastases and the prognosis, especially the prognosis after surgery. In this study, the SEER database was used to analyze the predictive effect of PAP on the PCa prognosis and the disease progress. We found that patients with PAP-positive subtype showed easier metastases, larger tumor size, more localized tumor numbers, higher pathological grade, and shortened survival duration. Moreover, the PAP-positive patients also present decreased survival months even after the surgical treatment. Though it is already suggested that PAP is one of the important markers for the test of the prognosis and the PCa progress, it is still lacking the population-based survival outcomes and disease progress, to our knowledge. Moreover, the details of PCa progression and prognosis were firstly present in this analysis.

PAP was first reported by Gutman in 1938 that the increased levels of serum PAP was observed in patients with metastatic prostate cancer [12]. Shortly thereafter, it is established that PAP is a tumor marker for PCa. It is also suggested that elevated PAP should be paid great attention to though maybe no clinical evidence of metastasis happens [13, 14]. Moreover, in the following years, the investigators reported that survival duration was significantly shortened for patients with elevated serum PAP [15], which is not completely consistent with this research. In the cancer of other tissues, small intestine, pancreas, and bladder included, PAP may also lead to an increased level. Thus, the slight elevation of PAP may reflect cancers other than the prostate [15, 16]. In the cancer-specific survival (CSS) study, it is showed that when PAP concentration is <1.5 U/L, 1.5-2.4 U/L, and >2.5 U/L, the progression of prostate cancer is 93%, 87%, and 75% (*P* = 0.013), respectively, which shows better than the PSA test (<10 ng/mL, 10-20 ng/mL, and >20 ng/mL, progression of prostate cancer is 92%, 76%, and 83%, *P* = 0.393, respectively). However, the CSS study is only involved in 193 patients [17]. In this study, CSS is also analyzed based on a population-based study. However, PAP is still not sensitive for the early stage diagnosis of the PCa and PSA was first isolated in seminal plasma in 1971 and widely used as a screening marker since 1990s.

Although PSA has largely replaced PAP as an early-stage screening marker, PAP is still an important prognostic marker in advanced and metastatic PCa [18]. It has been reported that secretory PAP (sPAP) in osteoblastic bone metastases stimulated collagen synthesis and alkaline phosphatase expression of bone cells [18]. After that, it was demonstrated not only for the elevation of sPAP in advanced PCa but also the cellular PAP (cPAP) expression is also highly expressed in human PCa bone metastases and stimulates preosteoblastic proliferation and differentiation [19]. It is also reported that PAP derived from PCa cells directly stimulates bone mineralization [16]. Recently, as is known, PAP secreted by PCa cells in osteoblastic bone metastases increases the RANK/RANKL/OPG system and plays a critical role in the vicious interaction between cancer and bone cells. Thus, the inhibition of sPAP may be a choice for PCa osteoblastic bone lesions [10]. Interestingly, it is reported that 88% of castration-resistant PCa (CRPC) bone metastases express prostatic acid phosphatase (PAP) in bone metastasis and there exists no significant difference between the osteoblastic and osteolytic lesions [16]. This may be due to the various kinds of factors involved in the osteolytic lesions. While PAP appears to drive the osteoblastic response, it can be moderated or negated by the other osteolytic factors in the microenvironment.

It is noteworthy that PAP is treated as a useful antigen for prostate cancer therapy. The vaccine treatment, sipuleucel-T, is used in clinic work. It is also the first vaccine for cancer treatment. This FDA-approved therapy is based on the idea that over 95% of prostate cancer cells express PAP specifically [20]. PAP is used to be fused with granulocyte-macrophage colony-stimulating factor (GM-CSF) thus presented to antigen-presenting cells (APCs) which are collected from the patient. These activated APCs are then introduced to the patient for induction of T cells in vivo and T cells are activated and attack prostate cancer cells in patients [15]. This vaccine treatment resulted in a 4.1-month longer median overall survival compared with placebo with a reduction in the risk of death [21].

Because of these recent applications of PAP, the PAP study returns to the view in the clinic work. In this study, PAP-positive patients present the higher metastasis rates, the larger tumor sizes, more localized tumor numbers, and higher pathological grade. The survival duration is also shortened in the PAP-positive group as was reported previously, which suggests a prognosis effect of PAP towards PCa. Interestingly, it is the first time to observe that the survival duration is still decreased in the PAP-positive group even after the surgery treatment. In this way, whether other treatment should be used before surgery for the PAP-positive individuals is still worth studying. Moreover, PAP is argued to be a good choice for the prognosis caused by PCa specifically but not other lethal factors, as is resulted from the COX regression study. Some even reported that the elevated serum pretreatment PAP levels were considered to be a relative contraindication to surgery [14, 18]. In our study, however, the survival months of surgical patients is still longer even in the PAP-positive group (95 (144-168) vs. 48 (118-156), *P* < 0.001, shown in Figure 2). Thus, surgery treatment is still needed for the PAP-positive PCa patients, and it should be paid attention to that these individuals may present a poorer outcome than the negative ones. In this way, it strongly suggest that serum PAP should be reused in the clinic work for the detection of the disease progress and the prognosis.

## 5. Limitations

No serum PSA level is available as the NCI removed PSA data from SEER after the “substantial number of registry-reported PSA values were incorrect” [22]. In this research, no exact PAP level was present as the method used for detection is varied and in SEER only qualitative results were present in this database. As is reported by Andras G. Foti et al. in 1977 [23], there are different methods and the relative reference value according to different methodologies. Thus, only PAP+ or PAP- were present in this study according to the SEER database. As the median age of all patients in the study is 68 (61-74), the proportion alive in the survival curve is approaching to zero after approximately 200 months. Thus, no significant differences are observed in the long-term survival analysis. Moreover, as the data were only available between 1998 and 2003 and the other data were substituted by PSA test for the PCa early-stage screening, more researches may be needed in the future study, especially for some high quality prospective clinic study.

## 6. Conclusions

In conclusion, despite of these limitations, our study provides insight into the PAP prediction in PCa patients in the United States. It lends support to the consideration of testing PAP for the PCa progress and the prognosis. Is this indicating the renewal of PAP?

## Figures and Tables

**Table 1:** Incidence proportion and median of patients with PAP-positive (PAP+) and PAP-negative (PAP-).

	PAP+ median (IQR)	No. (%) 5184, 100	PAP- median (IQR)	No. (%) 3161, 100	Total	*P* value
Age at diagnosis (y)						
30-50	48 (45-50)	161, 3.11	48 (46-50)	131, 4.14	292	0.365
51-70	63 (58-67)	2857, 55.11	63 (58-67)	1927, 60.96	4784	0.968
71-85	76 (73-79)	1972, 38.04	76 (73-78)	1037, 32.81	3009	^∗^0.025
≥86	88.5 (87-91)	194, 3.74	88 (87-90)	66, 2.09	260	0.071
Total		5184, 100		3161, 100		
Race						
White	NA	3838, 74.73	NA	2371, 75.41	6209	NA
Black	NA	1021, 19.88	NA	452, 14.38	1473	
Asian	NA	259,5.04	NA	304,9.67	563	
American Indian	NA	18, 0.35	NA	17, 0.54	35	
Total	NA	5136, 100	NA	3144, 100	8280	
*T*						
*T*_1_	NA	1362, 42.30	NA	791, 38.44	2153	^∗^0.03
*T*_2_	NA	1229, 38.17	NA	855,41.55	2084	
*T*_3_	NA	440,13.66	NA	297,14.43	737	
*T*_4_	NA	189, 5.87	NA	115,5.9	304	
Total	NA	3220, 100	NA	2058, 100	5278	
*N*						
*N*_0_	NA	3804, 98.81	NA	2605, 99.09	6409	^∗∗^0.005
*N*_1−3_	NA	46, 1.19	NA	24, 0.91	38	
Total	NA	3850, 100	NA	2629, 100	6479	
*M*						
*M*_0_	NA	4450, 89.07	NA	2974, 95.94	7424	^∗∗^<0.001
*M*_1_	NA	546, 10.93	NA	126,4.6	672	
Total	NA	4996, 100	NA	3100, 100	8096	
Invasion						
No invasion	NA	4185, 3.92	NA	2878, 92.87	7063	^∗∗^<0.001
Invasion beyond capsule	NA	242,4.85	NA	94, 3.03	336	
Distant metastasis	NA	560, 11.23	NA	127, 4.10	687	
Total	NA	4987,100	NA	3099, 100	8086	
Grade						
1	NA	174,3.66	NA	143,4.66	317	
2	NA	3052, 64.23	NA	2197, 71.56	5249	^∗∗^<0.001
3	NA	1498, 31.52	NA	711,23.16	2209	
4	NA	28, 0.59	NA	19, 0.62	47	
Total	NA	4752, 100	NA	3070, 100	7822	
Number of localized tumors	1.0 (1.0-1.0)	5184,100	1.0 (1.0-2.0)	3161, 100	8345	^∗∗^<0.001
Survival months (all-cause)	134.0 (63.0-161.0)	4761, 100	143.0 (88.0-166.0)	2821, 100	7582	^∗∗^<0.001
Survival months (prostate cancer-specific)	47.00 (16.00-90.00)	963, 100	93.00 (52.75-132.00)	962, 100	1925	^∗∗^<0.001
Survival months (postsurgical)	144.0 (95.0-168.0)	1642, 100	152.0 (125.8-172.0)	1118, 100	2760	<0.001

To value the PAP+/- ratio of each subgroups, the total effective patient number of each group is calculated. As the data were measurement data and ranked data, the Mann-Whitney Sum test was utilized. Abbreviations: + denotes positive; − denotes negative; PAP: prostatic acid phosphatase; TNM: tumor-node-metastasis; No.: number; %: percent; ^∗^*P* ≤ 0.05; ^∗∗^*P* ≤ 0.01.

**Table 2:** The multivariable logistic regression for the presence of metastases at diagnosis of prostate cancer.

	Pt. no.			Multivariable logistic regression analysis Hazard Ratio (95% CI)			
	Localized	With invasion	With metastasis	Among pt. with invasion	*P* value	Among pt. with metastasis	*P* value
Age at diagnosis (y)							
30-50	256	13	17	1 (Reference)	NA	1 (Reference)	NA
51-70	4217	185	283	0.86 (0.49-1.54)	0.62	1.01 (0.61-1. 68)	0.97
71-85	2438	125	325	1.01 (0.56-1.81)	0.97	2.01 (1.21-3. 32)	^∗∗^0.01
≥86	152	13	62	1.68 (0.76-3.73)	0.20	6.14 (3.46-10.89)	^∗∗^<0.01
Race							
White	5303	238	478	1 (Reference)	NA	1 (Reference)	NA
Black	1184	79	157	1.49 (1.14-1.93)	^∗∗^<0.01	1.47 (1.22-1. 78)	^∗∗^<0.01
Asian	495	15	42	0.68 (0.40-1.15)	0.15	0.94 (0.68-1.31)	0.72
American Indian	21	4	8	4.24 (1.45-12.46)	^∗∗^0.01	4.23 (1.86-9.59)	^∗∗^<0.01
Grade							
1	299	6	6	1 (Reference)	NA	1 (Reference)	NA
2	4880	139	146	1.42 (0.62-3.24)	0.41	1.49 (0.65-3.40)	0.34
3	1639	173	315	5.26 (2.31-11.98)	^∗∗^<0.01	9.58 (4.23-21.68)	^∗∗^<0.01
4	26	3	16	5.75 (1.36-24.33)	^∗^0.02	30.67 (11.06-85.07)	^∗∗^<0.01
Number of localized tumors							
1	6218	304	648	1 (Reference)	NA	1 (Reference)	NA
≥2	845	32	39	0.81 (0.61-1.07)	0.13	0.74 (0.61-0. 91)	^∗∗^<0.01
PAP							
-	2878	242	127	1 (Reference)	NA	1 (Reference)	NA
+	4185	94	560	1.78 (1.39-2.26)	^∗∗^<0.01	3.03 (2.49-3.70)	^∗∗^<0.01

The multivariable logistic regression was used to determine whether age and race were associated with the presence of invasion and metastases at diagnosis; other variables in the model included pathological grade and number of localized tumors. The likelihood ratio test (LRT) is used in the multivariable logistic regression analysis. Abbreviations: PAP: prostatic acid phosphatase; y: years; Pt. no.: patient number; ^∗^*P* ≤ 0.05; ^∗∗^*P* ≤ 0.01.

**Table 3:** The multivariable Cox regression for all-cause mortality and prostate cancer-specific mortality among PCa patients and postsurgical dead.

	All-cause mortality			Prostate cancer-specific mortality		
	Pt. no.	Hazard Ratio (95% CI)	*P* value	Pt. no.	Hazard Ratio (95% CI)	*P* value
Age at diagnosis (y)						
30-50	292	1 (Reference)	NA	285	1 (Reference)	NA
51-70	4784	2.88 (1.84-4.51)	^∗∗^<0.01	4487	2.16 (1.24-3.77)	^∗∗^0.01
71-85	3009	7.57 (4.82-11.89)	^∗∗^<0.01	2662	6.09 (3.47-10.69)	^∗∗^<0.01
≥86	260	23.43 (14.17-38.73)	^∗∗^<0.01	228	17.34 (8.63-34.83)	^∗∗^<0.01
Race						
White	6209	1 (Reference)	NA	5649	1 (Reference)	NA
Black	1473	1.33 (1.17-1.50)	^∗∗^<0.01	1386	1.46 (1.20-1.77)	^∗∗^<0.01
Asian	563	0.85 (0.72-1.01)	0.06	529	0.81 (0.63-1.05)	0.11
American Indian	35	2.00 (0.64-6.27)	0.24	34	1.25 (0.17-9.05)	0.82
*T*						
*T*_1_	2153	1 (Reference)	NA	1954	1 (Reference)	NA
*T*_2_	2084	0.95 (0.86-1.05)	0.31	1927	0.96 (0.82-1.13)	0.64
*T*_3_	737	0.59 (0.48-0.72)	^∗∗^<0.01	692	0.66 (0.49-0.89)	^∗∗^0.01
*T*_4_	304	0.98 (0.76-1.26)	0.88	276	0.87 (0.59-1.28)	0.49
*N*						
*N*_0_	6409	1 (Reference)	NA	5898	1(Reference)	NA
*N*_1_	38	2.85 (1.68-4.85)	^∗∗^<0.01	36	5.01 (2.65-9.46)	^∗∗^<0.01
*N*_2_	27	1.50 (0.80-2.83)	0.21	25	3.10 (1.45-6.65)	^∗∗^<0.01
*N*_3_	5	3.82 (1.41-10.32)	^∗∗^0.01	5	2.29 (0.32-16.53)	0.41
*M*						
*M*_0_	7424	NA	NA	6857	NA	NA
*M*_1_	672	NA	NA	590	NA	NA
Grade						
1	317	1 (Reference)	NA	281	1 (Reference)	NA
2	5249	0.79 (0.65-0.96)	^∗^0.02	4847	0.81 (0.60-1.10)	0.18
3	2209	1.20 (0.98-1.48)	0.08	2025	1.41 (1.03-1.92)	^∗^0.03
4	47	2.39 (1.20-4.73)	^∗∗^0.01	39	2.35 (0.83-6.68)	0.11
Number of localized tumors						
1	6475	1 (Reference)	NA	6475	1 (Reference)	NA
≥2	1870	1.88 (1.71-2.06)	^∗∗^<0.01	1187	1.89 (1.62-2.21)	^∗∗^<0.01
Location						
Localized	7063	1 (Reference)	NA	652	1 (Reference)	NA
Invasion	336	2.09 (1.64-2.66)	^∗∗^<0.01	1 311	2.21 (1.54-3.18)	^∗∗^<0.01
Metastasis	687	6.48 (3.42-12.30)	^∗∗^<0.01	606	9.52 (4.47-20.24)	^∗∗^<0.01
PAP						
-	3161	1 (Reference)	NA	2879	1 (Reference)	NA
+	5184	1.04 (0.95-1.14)	0.42	4783	2.87 (2.48-3.32)	^∗∗^<0.01
Surgery						
Y	2760	1 (Reference)	NA	2553	1 (Reference)	NA
N	5585	1.12 (1.00-1.26)	^∗^0.05	5109	1.09 (0.91-1.30)	0.36

The multivariable Cox regression was performed to identify covariates associated with increased all-cause mortality using the same variables as in the logistic regression model described. In the Cox regression model, enter method was used. Abbreviations: Pt. no.: patient number; y: years; TNM: tumor-node-metastasis; Y: yes; N: no; ^∗^*P* ≤ 0.05; ^∗∗^*P* ≤ 0.01.

## Data Availability

The data used to support the findings of this study are available from the corresponding author upon request.
